# Management of musculoskeletal pain in patients with idiopathic pulmonary fibrosis: a review

**DOI:** 10.48101/ujms.v127.8739

**Published:** 2022-07-11

**Authors:** Svetlana Kašiković Lečić, Jovan Javorac, Dejan Živanović, Aleksandra Lovrenski, Dragana Tegeltija, Jelena Zvekić Svorcan, Jadranka Maksimović

**Affiliations:** a Institute for Pulmonary Diseases of Vojvodina, Sremska Kamenica, Serbia; b University of Novi Sad, Faculty of Medicine, Department of Internal Medicine, Novi Sad, Serbia; c College of Vocational Studies for the Education of Preschool Teachers and Sports Trainers, Department of Biomedical Sciences, Subotica, Serbia; d University of Novi Sad, Faculty of Medicine, Department of Pathology, Novi Sad, Serbia; e Special Hospital for Rheumatic Diseases, Novi Sad, Serbia; f University of Novi Sad, Faculty of Medicine, Department of Medical Rehabilitation, Novi Sad, Serbia; g Institute of Epidemiology, Faculty of Medicine, University of Belgrade, Belgrade, Serbia

**Keywords:** IPF, dyspnea, cough, pain, treatment

## Abstract

Idiopathic pulmonary fibrosis (IPF) is a chronic, progressive, fibrotic, and fatal interstitial lung disease (ILD) of unknown etiology that primarily affects the elderly. Patients with IPF suffer from a heavy symptom burden and usually have a poor quality of life. Dyspnea and dry cough are predominant symptoms of IPF. Although pain is not considered one of the main symptoms of IPF, it can occur for a variety of reasons, such as hypoxia, coughing, muscle and nerve damage, deconditioning, and steroid use. The prevalence of pain in IPF patients varies greatly, ranging from around 30 to 80%, with the prevalence being estimated mostly among patients in the end-of-life period. It manifests itself in the form of muscle pain, joint discomfort, or back and chest pain. Approaches to the treatment of chronic musculoskeletal pain in patients with IPF include pharmacological and non-pharmacological measures that are also important to optimize the treatment of other symptoms (dyspnea and cough) and the optimal treatment of comorbidities. Given the scarcity of data on this symptom in the literature, this article summarizes what is currently known about the etiology and treatment of musculoskeletal pain in IPF.

## Introduction

Idiopathic pulmonary fibrosis (IPF) is a chronic, progressive fibrosing interstitial pneumonia of unknown origin with a median survival of 3–5 years after diagnosis, which is similar or even worse than the time course of many malignancies (1). Lung fibrosis is defined by the replacement of the normal, compliant, elastin-rich lung extracellular matrix, with an aberrant matrix rich in fibrillar collagen (2). The pattern of alveolar lesions in IPF is consistent with the usual interstitial pneumonia (3). One of the defining features of IPF is its unpredictable clinical course, which can range from long periods of clinical stability to acute exacerbations with rapid decompensation (4). For some patients, the disease progresses rapidly. Others may experience a slow progression of the disease, in which their lung function remains stable for an extended period of time, and they live a considerably longer life (5). The majority of patients with IPF develop symptoms between the ages of 50 and 70, while IPF is seldom diagnosed in those under 50 years of age (5, 6). Patients with a long-term IPF may have a heavy symptom burden with breathing difficulties (dyspnea), a persistent dry cough and fatigue being the most prominent (7–9). Even though gradual, unintended weight loss, muscular pain (myalgias), or joint pain (arthralgias) are not very common and may indicate an alternative diagnosis (6), these symptoms may occur and contribute to a lower quality of life in patients with IPF.

At rest, IPF patients usually demonstrate restrictive pulmonary physiology with decreased forced vital capacity and total lung capacity, accompanied by impaired gas exchange as measured by the diffusion capacity of the lung for carbon monoxide (2). As the disease advances, lung compliance declines and lung volumes decrease (2, 5). Abnormal pulmonary gas exchange, inefficient breathing mechanics, exercise-induced hypoxemia, circulatory impairments, and respiratory and skeletal muscle dysfunction are all factors that impede exercise performance of patients with IPF, as they do in other chronic pulmonary diseases (10). Changes in lung mechanical characteristics, anomalies in the lung vasculature, and diffusion dysfunction all contribute to the early onset of chronic arterial hypoxemia during exercise and the later onset of chronic arterial hypoxemia at rest in IPF (2).

The aim of IPF treatment is to slow disease progression, reduce the frequency of acute exacerbations, increase survival, reduce symptoms, and improve overall health-related quality of life (HR-QoL). Currently, two anti-fibrotic medications are approved for the treatment of IPF (pirfenidone and nintedanib) (8). They can slow disease progression but provide inadequate symptom relief (11). Similar to other progressive pulmonary diseases, IPF has psychological, social, and existential consequences, affecting overall quality of life (12). Alleviating symptoms and improving HR-QoL in patients with IPF are often a major challenge for clinicians. In a small minority of patients, lung transplantation is an option that can increase survival and improve health-related wellbeing (11, 13).

Because IPF is more common in older patients and smokers, these patients are more likely to have multiple comorbidities, such as chronic obstructive pulmonary disease (COPD), emphysema, bronchiectasis, sleep apnea, pulmonary hypertension, lung cancer, cardiovascular diseases, pulmonary embolism, gastro-esophageal reflux disease (GORD), depression, anxiety (14). These comorbidities must be carefully examined since they frequently increase symptom burden and may have a negative impact on functional status, quality of life, and prognosis (15). Treatment of comorbidities can be challenging: there is no evidence to treat pulmonary hypertension in IPF (16, 17), gastro-esophageal reflux therapy is a matter of debate (18), and lung cancer treatment is a major problem (19). Other comorbidities are treated similarly to patients without IPF (15).

The management of IPF is multifaceted, requiring collaboration among members of the healthcare team, family members, and caregivers to provide patient education and support, as well as management of symptoms, comorbidities, and palliative care (13). Musculoskeletal pain, while not the most common symptom, has a significant impact on the overall quality of life of patients with IPF. Therefore, we conducted a review of literature to summarize what is currently known about the etiology, frequency, and treatment of musculoskeletal pain in IPF.

## Materials and methods

Using selected keywords (‘idiopathic pulmonary fibrosis’, ‘interstitial lung diseases’, ‘musculoskeletal pain’, ‘treatment’, ‘dyspnea’, ‘cough’, ‘quality of life’, and ‘palliative care’), we searched bibliographic databases PubMed, Medline, and Google Scholar for available meta-analysis, literature, and systematic reviews, as well as clinical studies where the musculoskeletal pain in patients with IPF and ILDs, in general, was analyzed. The research included relevant literature sources, only in English, that had been published within a 12-year period (2000–2022) and that met the following inclusion criteria: number of citations, year of publication, and educational significance of available information in a particular manuscript. All identified studies are presented and discussed in a non-selective manner. A total of 91 bibliographic sources were used in writing this paper. To the best of our knowledge, this is the first literature review describing research into musculoskeletal pain in IPF.

## Musculoskeletal pain in patients with IPF

Patients report that dyspnea, coughing, and fatigue have the greatest impact on their daily lives (7). Although pain is not considered one of the main symptoms of IPF, it can occur in some patients as chronic or frequent pain. Studies that evaluated the prevalence of pain in patients with IPF, its localization and mechanism of development, as well as modalities of pain management, are rare in the literature. The results they obtained were frequently inconsistent, indicating that pain in IPF is still understudied, and that future research should focus more on this symptom of the disease, which can contribute to the patient’s lower overall quality of life.

### The prevalence and localization of musculoskeletal pain in IPF

The prevalence of pain in IPF patients has been investigated in a small number of studies, and the results vary, with estimates ranging from 30 to 80%. The variations in the results can be explained by the different methodological approaches used in the research, as well as the fact that the patients in the studies were at different stages of their disease. The majority of research on pain in IPF included patients in their end-of-live period. Thus, in a study conducted by Rajala et al. (20) and Ahmadi et al. (21), symptoms during the last week of patient’s life were evaluated, and pain was found in 31 and 51% of patients, respectively. In the retrospective assessment of patients with a progressive idiopathic fibrotic ILD, including IPF, generalized pain was found in 9%, while chest pain was reported by 29% of patients (22). In another study by Rajala et al. (23), with a more general IPF cohort of patients (the average duration of IPF was 3.9 years), pain in movement was reported by 82% of patients, whereas pain in rest occurred in 66%. The subjects of this study localized pain in their chest, head, and neck; upper limbs; stomach; back; pelvis; and lower limbs, or they stated that pain was extensive. One-third of patients complained of chest pain, which showed linear relationship with the severity of dyspnea as measured by the modified Medical Research Council score. Another study also found a correlation between pain intensity and dyspnea severity (24), while this relationship was not found in others (25). These variations could be attributed to the fact that different study population were at different stages of disease.

### Etiology of musculoskeletal pain in IPF

Many patients cannot understand how a lung disease, such as IPF, can manifest into pain (often in the form of muscle pain, joint discomfort, or a sore back and chest). Some of the underlying causes of pain in patients with IPF are as follows:

- Lack of oxygen (hypoxia)

Patients with advanced IPF develop hypoxia (reduction of oxygen levels in cells and tissues) due to multiple physiologic derangements, including diffusion limitation, ventilation-perfusion mismatching, and abnormalities of the pulmonary vasculature (2). Hypoxia may contribute to metabolic (lactic) acidosis in muscles, which can cause pain in the joints, muscles, and other parts of the body (10, 26). Hypoxia during exercise impairs maximal workload and endurance time (27).

- Coughing

Coughing is common in patients with IPF (4, 27–29). The pathogenesis of cough in IPF is multifactorial and influenced by mechanical, biochemical, and neurosensory changes in the lungs, as well as patient’s comorbidities, such as COPD, GORD, obstructive sleep apnea, and cardiovascular diseases (28). Chronic cough is associated with impairments of several different aspects of the patient’s life, including poor sleep quality, limited exercise ability, decreased social interactions, and, eventually, musculoskeletal pain (28, 30–32). However, there is a lack of information on the precise mechanisms by which cough causes musculoskeletal pain in patients with IPF or ILD in general. Several hypotheses can be proposed. It is known that both the inspiratory and expiratory respiratory muscles are actively involved in the coughing process, as well as that extreme changes in intrathoracic pressure occur during this process due to the active contraction of these muscles (33). Chronic dry coughing and cough paroxysms, associated with dyspnea, may exhaust the respiratory muscles during the expansion and contraction of the thoracic cavity (34), leading to soreness in the shoulders, chest, and upper back. Sometimes, patients feel pain due to ribs injury caused by coughing (35).

- Muscle and nerve damage

Another and likely less common reason that can cause pain is muscle and nerve damage. Respiratory and limb muscle dysfunction can be caused by several different mechanisms, such as chronic lack of oxygen in the blood, inflammatory, and oxidative stress in the muscle; chronic corticosteroid administration; physical inactivity; ageing; and malnutrition (34). These claims are supported by the findings of several studies, where the force of quadriceps femoris muscle (36) or rectus femoris muscle (37) was reduced in patients with IPF. Chronic hypoxia may potentially cause nerve injury, but additional pathways for nerve damage have not been thoroughly examined. Given the prevalence of diabetes mellitus among IPF patients (38), as well as the effects of diabetes on the neurological system, the importance of this comorbidity in the management of pain in IPF patients must be considered.

- Deconditioning

Deconditioning or the reduced functional capacity of the musculoskeletal system can develop due to the disease. As IPF progresses, more and more of the lung tissue becomes scarred. The increased scarring leads to increased shortness of breath or breathlessness during activity. Patients naturally reduce their activity level and become deconditioned over time. Avoiding physical activities that provoke dyspnea and fatigue may be an important key factor of physical deconditioning and exercise intolerance (34). This can lead to muscle wasting, weakness, stiffness, and pain or discomfort during physical activity (39).

- Application of glucocorticoids

Guidelines recommend high-dose corticosteroid treatment in patients with acute exacerbations of IPF, despite unproven benefit (11, 40). Sometimes, corticosteroids are used for other reasons in patients with IPF (treatment of cough, dyspnea, and comorbidities). Corticosteroid-induced myopathy is the most common type of drug-induced myopathy, with an estimated incidence of 50–60% among patients who have been using corticosteroids for an extended period of time (41). It can affect both respiratory and peripheral muscles, resulting in muscle weakness and loss of muscle mass, accompanied sometimes with pain, cramps, or tightening sensation in the muscles (41, 42). The severity of corticosteroid-induced myopathy is determined by the type of steroid used, the treatment duration, the dose, and the treatment regimen, with repeated burst treatment effects being worse than those obtained with a continuous treatment with the same dose (43).

- Other painful conditions

Some people diagnosed with IPF may experience pain due to other coexisting medical conditions, such as arthritis. Sometimes, digital clubbing (the tips of the fingers or toes become rounded and enlarged, resembling drumsticks) can be painful to some patients. Digital clubbing occurs due to a chronic lack of oxygen in the blood (44). The presence of associated cardiovascular diseases can cause and intensify the chest pain in patients with IPF (45). IPF is also linked to GORD, which can cause discomfort or burning in the chest (46, 47).

Chronic pain has biological, psychological, and societal consequences. IPF patients who suffer from chronic pain, like any other chronic pain patient, may be more prone to illness and injury, as well as depression, anxiety, and social isolation; all of which contribute to a lower quality of life (48). Pain can interfere with the patients’ participation in a pulmonary rehabilitation program (49).

### How to treat pain associated with IPF?

In everyday clinical practice, patients may not associate musculoskeletal pain with their lung disease, so they may not report this symptom to their physician. Thus, clinicians should be encouraged to be more vigilant in asking patients about all symptoms, including pain, during examinations. Early detection, assessment, and treatment of symptoms related to disease progression, such as pain, are important. Palliative care can provide relief from the painful symptoms and stress that goes along with having IPF and can help improve quality of life of patients and their families (1, 4). A variety of pharmacologic and non-pharmacologic therapies are available to treat pain associated with IPF.

#### Pharmacologic treatment options

There is no therapy that is specifically designed to treat pain in IPF patients. The majority of treatments target other pain-related symptoms of IPF, such as cough, manage comorbidities that are associated with pain, or standard analgesics are used (primarily opioids, with no available literature data on the effects of non-steroidal anti-inflammatory drugs, NSAIDs, or paracetamol).

- Coughing reduction treatments

When cough is present in IPF, it is severe and difficult to treat as it is often refractory (50). Cough treatment may alleviate the pain associated with continued hacking. Cough associated with IPF can be due to underlying lung disease or comorbidities. The first step in managing chronic cough in IPF is to rule out any potential comorbidities (27). The most common causes of chronic cough are asthma, postnasal drip syndrome, and acid reflux from the stomach (51). The less common causes include (viral) infections, eosinophilic bronchitis, pleural diseases, and the use of drugs such as angiotensin-converting enzyme inhibitors. Treatment of comorbidities must be implemented before chronic cough may be considered directly linked to the underlying disease – IPF (49, 52). A recent multicenter prospective observational study of pirfenidone reported decreased objective cough without significant changes in quality of life (53). The beneficial effects of pirfenidone on cough in IPF patients were confirmed in several other studies (54, 55). There are limited data on nintedanib’s effects on cough in IPF patients. In the INBUILD trial in patients with progressive fibrosing interstitial lung diseases, results suggested that nintedanib may prevent worsening of cough (56), while no clinically significant effect of nintedanib on cough was observed in INPULSIS trials with IPF patients (57). Conventional antitussive therapy is frequently ineffective (58). Among centrally active antitussives, opioids (codeine and morphine) are the most commonly used for this purpose, but with limited efficacy and often systemic side effects (50). The opinion that codeine is an effective cough suppressant is not supported by the available evidence (59). Antitussives have been studied in IPF only a few times and on a small basis. Despite the fact that thalidomide (60) and interferon-alpha (61) have been shown to improve cough in IPF patients, they are still not approved for this indication and are either extremely expensive for off-label use (thalidomide) or not commercially available (interferon-alpha). Because cough is one of the most prevalent symptoms reported by IPF patients and one of the factors contributing to their poor HR-QoL, it would be beneficial to incorporate cough outcome measures in future studies of new IPF medications. Based on therapeutic studies in the chronic non-IPF idiopathic cough population, future study should focus on medications that suppress the cough reflex, such as gabapentin, pregabalin amitriptyline, inhaled cromolyn sodium (PA101), or P2X3 inhibitors, which may provide cough alleviation in IPF as well (50, 62).

- Low doses of morphine

Opioids have been shown to be effective in reducing intensity of pain in a variety of chronic pain conditions (63). Individually titrated doses between 10 and 30 mg per day have showed to improve dyspnea, cough, and pain in patients with advanced IPF (64). In general, the lowest form of morphine tablet or equivalent is 5 mg, which may not be tolerated by the elderly. Because morphine is available in very low doses of 1 mg/mL in liquid form, clinician can start with minimal doses and gradually titrate over longer periods of time to monitor adverse drug reactions and treatment response (65). When prescribing these medications in the elderly, certain factors other than comorbidities should be considered, such as an increase in the pain threshold and a physiological decline in hepatic and renal function, which may affect the pharmacocinetic of analgesics, including the onset of action, elimination rate, and half-life of the drug (66). Opioid-related adverse effects (primarily constipation, nausea, dizziness, and somnolence) are well documented in the literature and should be managed conservatively (67). Even though the use of opiates to treat IPF symptoms is still controversial, it should be considered when the patients’ quality of life is severely impaired if there are no other treatment options available (62).

- NSAIDs and paracetamol

Non-steroidal anti-inflammatory drugs are commonly used to treat mild-to-moderate musculoskeletal pain (68). They alleviate the pain by decreasing the activity of cyclooxygenase enzymes and inhibiting prostaglandin synthesis, but they also increase the risk of gastrointestinal, cardiovascular, and renal adverse events (69). Another issue with their use is that due to comorbidities and contraindications, they are frequently ineffective in elderly and severely ill patients (70). Topical NSAIDs were found to be more effective than oral formulations in the management of pain in osteoarthritis, with fewer adverse events, but this effect was not seen in other conditions accompanied by musculoskeletal pain (71). Paracetamol is one of the most commonly used medications for pain and fever, with effective analgesic and antipyretic effects, and minor gastrointestinal, renal, and vascular adverse effects. Despite reports of paracetamol-associated acute liver injury, paracetamol remains a preferred analgesic, particularly for elderly and frail patients (70). In everyday clinical practice, it is evident that IPF patients typically use NSAIDs or paracetamol to relieve their musculoskeletal pain. However, we did not find any study in the literature that investigated the effect of these drugs on pain relief in IPF or any other ILD. Therefore, this is unarguably one of the issues that should be addressed in future research.

- Glucocorticoids

Currently, there is no evidence to support the use of high-dose steroids (62). When considering it as a possible treatment for pain in IPF, it should be taken into account that high doses of steroids have been shown to increase morbidity and mortality in IPF (58, 72), as well as their potential adverse effects. Oral corticosteroids have been shown to be effective in improving cough in one study of six IPF patients (58), with reduced cough symptoms on a visual analogue scale and reduced cough sensitivity to inhaled capsaicin and substance P. Low doses of prednisone are sometimes prescribed in daily practice for this purpose and are then gradually tapered if they are beneficial (22, 27), but this is not recommended in CHEST guideline and expert panel report on the treatment of ILD-associated cough (62). However, in some studies, no effects of corticosteroids on cough were reported (73), so the use of corticosteroids for this indication is still controversial and requires future investigations.

- Thalidomide

Thalidomide has been shown to reduce cough in IPF patients. This drug has anti-inflammatory and antiangiogenic effects, similar to currently used anti-fibrotic drugs (27), but with potentially severe side effects such as constipation, venous thromboembolism, skin rash, dizziness, malaise, and peripheral neuropathy (suggesting that it may also have effects on sensory nerves) (74). Thalidomide should not be considered a routine treatment for cough in IPF, even as a second-line therapy, until further evaluation of the benefit/risk ratio has been undertaken (60, 62).

- Supplemental oxygen therapy

As previously speculated, hypoxia that occurs during the course of IPF may lead to musculoskeletal pain in these patients. Therefore, supplemental oxygen therapy may alleviate pain in IPF patients (75). There are no studies in the literature that investigated the effects of supplemental oxygen therapy on pain reduction in IPF patients. The IPF clinical practice guidelines, on the other hand, make a strong recommendation for the use of long-term oxygen therapy (LTOT) in patients with advanced lung disease and with clinically significant resting hypoxemia (5). Supplemental oxygen therapy has been empirically shown to have some beneficial effects on cough, which may have an indirect impact on pain in these patients (22). Studies on oxygen use in IPF patients are generally limited, and there is no data demonstrating the benefit of supplemental oxygen therapy for all patients with IPF. Patients who may benefit from LTOT are classified as either those who benefit from oxygen at rest (will be treated with LTOT) or those who benefit from oxygen during exertion (75). For patients who are breathless on exertion and have a desaturation on exertion <90%, ambulatory oxygen should be considered if this leads to improved exercise capacity or reduced breathlessness. Oxygen administration is associated with decreased exertional dyspnea and improved exercise capacity by increasing cardiac output and arterial oxygen content (76, 77).

#### Non-pharmacologic treatment options

Given that pharmacological treatments for the management of pain in IPF patients are very limited, and often ineffective, non-pharmacologic interventions serve as an important therapeutic tool for symptomatic management. The most important non-pharmacological option is pulmonary rehabilitation, but other methods may be beneficial.

- Pulmonary rehabilitation

Pulmonary rehabilitation is recommended in the IPF guidelines as a possible supportive therapy (5). The main components of pulmonary rehabilitation are exercise training, breathing therapy, smoking cessation, education and motivation, nutritional interventions, and psychosocial support. These therapeutic components differ individually and are dependent on the patients’ specific health status and personal goals (78). The goals of pulmonary rehabilitation management in IPF include the following: optimizing alveolar ventilation and lung volumes and capacities; reducing the work of breathing; maximizing aerobic capacity and efficiency of oxygen transport; optimizing physical endurance and exercise capacity, as well as general muscle strength and thereby peripheral oxygen extraction. The role of pulmonary rehabilitation in pain reduction can be seen in these goals, as it influences certain mechanisms that lead to pain, such as tissue hypoxia, nerve and muscle damage, and deconditioning. Some studies have also found some benefits of pulmonary rehabilitation on cough reduction, which can indirectly also affect pain in IPF patients (79).

Exercise training is an important part of pulmonary rehabilitation in IPF, since it improves exercise performance and overall health (80). Skeletal muscle dysfunction is a cardinal feature of IPF and is associated with severe exertional dyspnea and fatigue, as well as pain and poor HR-QoL (39). Exercise reduces musculoskeletal pain by decreasing ion channel expression, increasing the expression of endogenous analgesic substances (neurotrophins) in exercising muscle, and changing local immune cell function (increased anti-inflammatory cytokines) (81). According to the American Thoracic Society and the European Respiratory Society, pulmonary rehabilitation with exercise training is recommended for chronic respiratory diseases including ILD and IPF, providing both short- and long-term benefits (82). A recent systematic review concluded that exercise-based pulmonary rehabilitation is a safe and effective treatment for IPF patients, suggesting its prescription as standard care for these patients (83). Pulmonary rehabilitation improves sustained submaximal exercise capacity and anaerobic threshold in patients with IPF, reduces exercise-induced lactic acidosis, and increases oxidative enzyme activity in peripheral muscles (84), all of which can reduce the intensity of musculoskeletal pain.

Exercise training may be challenging to implement in IPF due to severe signs and symptoms experienced by the patients, particularly during exercise. Accordingly, several studies found that mild-moderate IPF patients adapted better to exercise training programs than severe IPF patients (85). Therefore, the emphasis in pulmonary rehabilitation is on supervised and safe exercise to improve functional capacity, as well as instructing and motivating patients to maintain home-based follow-up training modalities to retain the benefits of pulmonary rehabilitation (86).

Interventional exercise training studies in IPF exhibit variability in the training protocols and research methods used (28). The majority of them combined aerobic exercise (walking or cycling or both) with resistance and flexibility exercises for peripheral skeletal muscles (87–91). Some programs also included respiratory muscle training or breathing exercises (87, 88, 90). Breathing and balance exercises can also be beneficial for patient’s overall health (87).

The majority of the studies followed the established COPD guidelines for exercise training in the pulmonary rehabilitation program. These guidelines might be less appropriate for IPF due to different pathophysiological mechanisms of exercise limitation and, therefore, may not provide optimal exercise stimuli and adaptation to training (82). Further research in large randomized controlled trials should address different training modalities in order to optimize the exercise training programs for IPF. It should be carefully considered whether supervised exercise training-based pulmonary rehabilitation programs should be recommended as the standard of care for IPF patients.

- Other non-pharmacologic treatment options

Patient education should be prioritized among other non-pharmacological pain treatments. Patients’ central pain processing can be altered by educating them about pain mechanisms and challenging maladaptive pain cognitions and/or behaviors. The goal of education and cognitive-behavioral therapy is to change beliefs and behaviors that contribute to pain, fear, catastrophizing, and anxiety (81). Non-pharmacological interventions such as speech pathology therapy and Physiotherapy and Speech and Language Intervention have also been shown to be effective in reducing chronic cough, potentially impacting pain in IPF patients (62).

## Conclusion

To the best of our knowledge, this is the first narrative review that had addressed musculoskeletal pain in IPF patients. We could not find any original research that focused only on pain in these patients, as well. Musculoskeletal pain, on the other hand, is reported by one-third to two-thirds of IPF patients, indicating that it is a clearly understudied and underestimated problem. Alleviating symptoms and improving HR-QoL in IPF are often major challenges to treating clinicians. Because current therapeutic options are limited, patients with IPF require multidisciplinary care that includes disease education, communication, symptom management, and supportive care. Non-pharmacological interventions, such as pulmonary rehabilitation, present the cornerstone for symptom management. Pharmacological therapies (medications) can also alleviate severe symptoms, such as dyspnea, cough, and pain, but there is no single agent that is specifically designed to treat pain in IPF. Another point to emphasize is the presence of comorbidities, which can influence symptoms and should be considered for pain treatment. Further research is needed to get a better estimate of the prevalence, underlying mechanisms, severity, and pain predictors in IPF, as well as the pharmacological and non-pharmacological treatment options.

## Disclosure statement

The authors report no conflict of interest.

## Funding

There was no funding involved.

## Notes on contributors

***Svetlana Kašiković Lečić***, MD, PhD, is an internal medicine specialist and pulmonologist in the Institute for Pulmonary Diseases of Vojvodina, Sremska Kamenica, Serbia, and an associate professor in the Dept. of Internal Medicine, University of Novi Sad, Serbia.

***Jovan Javorac***, MD, PhD Candidate, is an internal medicine resident in the Institute for Pulmonary Diseases of Vojvodina, Sremska Kamenica, Serbia, an associate researcher in the Dept. of Internal Medicine, University of Novi Sad, Serbia, and a teaching assistant in the Dept. of Biomedical Sciences, College of Vocational Studies for the Education of Preschool Teachers and Sports Trainers, Subotica, Serbia.

***Dejan Živanović***, RN, PhD, is a clinical nursing specialist and teaching assistant in the Dept. of Biomedical Sciences, College of Vocational Studies for the Education of Preschool Teachers and Sports Trainers, Subotica, Serbia.

***Aleksandra Lovrenski***, MD, PhD, is a pathologist in the Institute for Pulmonary Diseases of Vojvodina, Sremska Kamenica, Serbia, and an assistant professor in the Dept. of Pathology, University of Novi Sad, Serbia.

***Dragana Tegeltija***, MD, PhD, is a pathologist in the Institute for Pulmonary Diseases of Vojvodina, Sremska Kamenica, Serbia, and an assistant professor in the Dept. of Pathology, University of Novi Sad, Serbia.

***Jelena Zvekić Svorcan***, MD, PhD, is a physical medicine and rehabilitation specialist and rheumatologist in the Special Hospital for Rheumatic Diseases, Novi Sad, Serbia, and an assistant professor in the Dept. of Medical Rehabilitation, University of Novi Sad, Serbia.

***Jadranka Maksimović***, MD, PhD, is an epidemiologist in the Institute of Epidemiology, Belgrade, and an associate professor in the Faculty of Medicine, University of Belgrade, Belgrade, Serbia.

## ORCID

Svetlana Kašiković Lečić https://orcid.org/0000-0002-4151-6121

Jovan Javorac https://orcid.org/0000-0002-8567-8974

Dejan Živanović https://orcid.org/0000-0001-8232-9368

Aleksandra Lovrenski https://orcid.org/0000-0002-5278-6194

Dragana Tegeltija https://orcid.org/0000-0002-9269-6644

Jelena Zvekić Svorcan https://orcid.org/0000-0002-3489-4351

Jadranka Maksimović https://orcid.org/0000-0002-8215-7399
